# Postcolumn Infusion as a Quality Control Tool for LC-MS-Based Analysis

**DOI:** 10.1021/jasms.2c00022

**Published:** 2022-04-28

**Authors:** Oskar González, Anne-Charlotte Dubbelman, Thomas Hankemeier

**Affiliations:** Analytical BioSciences and Metabolomics, Leiden Academic Centre for Drug Research, Leiden University, Einsteinweg 55, 2333 CC Leiden, The Netherlands

## Abstract

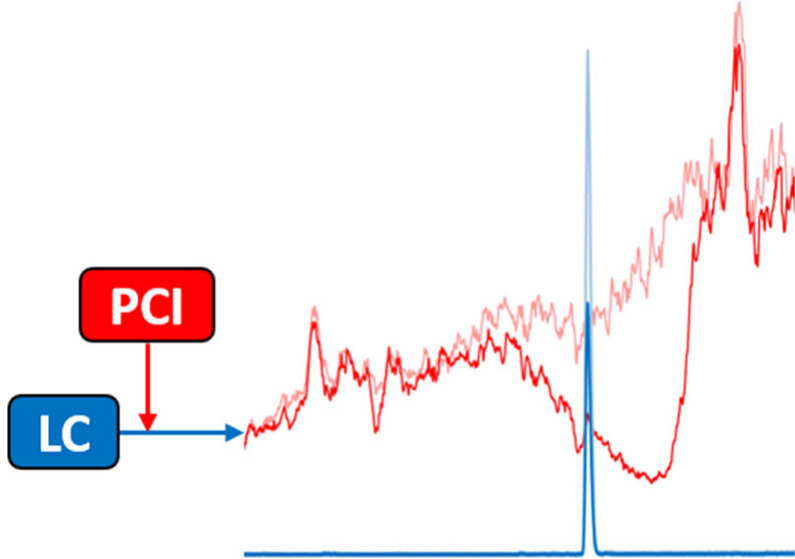

Postcolumn infusion has been widely used to study the matrix effect of analytical methods based on liquid chromatography coupled to mass spectrometry (LC-MS). Nevertheless, this methodology is usually only applied during a method development or validation. With this application note, we aim to demonstrate that the continuous use of postcolumn infusion can be also a very useful tool to monitor the quality of LC-MS analyses and easily detect flaws in the analytical method performance. Here we propose a protocol that can be transferred to other LC-MS platforms, and we show some real situations in bioanalysis in which postcolumn infusion proved to be extremely helpful in, for example, the evaluation of a sample treatment or the detection of unexpected sources of the matrix effect.

## Introduction

Matrix effect is defined as the effect of other compounds in the matrix, different than the analyte, on the quantification of the analyte.^1^ In liquid chromatography coupled to mass spectrometry (LC-MS), the ionization of the analyte can be affected by coeluting compounds (e.g., by influencing the droplet formation or the accessibility to charges).^2−4^ This phenomenon was traditionally overlooked, despite the huge impact it can have on the quantification. However, since some years ago, several approaches have been applied to avoid the negative impact of matrix effect (exhaustive sample treatment, improved chromatographic separation, use of labeled internal standards, alternative ionization methods, etc.),^5−7^ and its study has gained relevance. Indeed, nowadays, matrix effect is considered one of the main weaknesses of quantitative mass spectrometry.

While the determination of matrix effect is usually performed spiking the analytes in a blank matrix, from a qualitative point of view, the most common approach to study matrix effect is the postcolumn infusion approach proposed by Bonfiglio et al.^8^ Briefly, this methodology applies postcolumn infusion of the analytes while the analysis of an extracted blank sample or a solvent sample is running. The response of the analytes is monitored during the whole chromatogram in order to obtain the so-called matrix effect profiles. Then, the results obtained for the blank sample and the solvent sample are compared to look for the areas affected by matrix effect (either ion suppression or ion enhancement). One of the most remarkable aspects of this technique is that information about matrix effect is obtained at every retention time of the chromatogram. In this way, if few suitable model compounds are introduced by postcolumn infusion, the matrix effects that are affecting to several other compounds could be better understood. This is of special interest in fields like environmental research, toxicology, and metabolomics, when a high number of analytes is to be analyzed and/or when approaches such as the use of isotopically labeled internal standards show some limitations and postcolumn infusion can offer valuable information.

Although it is common to apply postcolumn infusion during a method development or validation,^9,10^ it is not usually applied to a real sample analysis or routine analysis. Considering that monitoring the matrix effect profiles can offer valuable information at any point of the chromatogram, the use of postcolumn infusion can be of great interest to improve the reliability of the analytical method. In this respect, it is advisible to use compounds with physicochemical properties similar to those of the analytes, since they are expected to have a similar ionization behavior but with a signal easily distinguishable from compounds present in the sample. Taking into account all these facts, isotopically labeled analogues are ideal candidates to study matrix effect profiles via postcolumn infusion, although more affordable compounds could also be used.^11^

In this Application Note, we explain the use of postcolumn infusion as a tool to monitor the quality of analytical methods and results based on LC-MS. With this aim, we use real-case situations in the field of bioanalysis that include the evaluation of the efficiency of the sample preparation and the detection of nonexpected sources of matrix effect. The methodology we propose here can be easily transferred to any other LC-MS method and does not increase the analysis time.

## Experimental Section

### LC-MS Conditions

The analytical method was developed for the analysis of more than 80 drugs in combination with metabolomic analysis, as has been described elsewhere.^12^ The platform consists of an Acquity UPLC (Ultra Performance Liquid Chromatography) system (Waters) coupled to a Synapt G2S HRMS (High Resolution Mass Spectrometer) instrument (Waters) with an electrospray ionization source operated in positive ionization mode (ESI+). The MS method consisted of a low collision energy function (2 eV) and a high collision energy function (ramp from 15 to 40 V) each with a scan time of 0.1 s. The collision energy was applied in the transfer cell keeping the collision energy of the trap cell constant at 4 eV. The ESI voltage was at 0.9 kV; the cone voltage was at 30 V, and source offset was at 80 V. The source temperature was set at 150 °C with desolvation temperature at 500 °C. Nitrogen was used for the cone, desolvation, and nebulizer gases with settings of 50 L/h, 1000 L/h, and 6 bar, respectively. Data were collected in the continuum mode with a scan range of 50–850 *m*/*z*, and the Time-of-Flight (TOF) detector worked in Resolution mode. A solution containing 0.1 mg/L leucine enkephalin (acetonitrile/water 50:50 with 0.1% v/v formic acid) and infused at a constant flow of 10 μL/min was used as the lock mass; a single point scan was collected every 10 s and averaged over three scans to perform mass correction (556.2771 *m*/*z*). The instrument was calibrated before analysis using sodium formate.

Chromatography was performed at 40 °C on a Waters Acquity UPLC HSS T3 column (1.8 μm, 2.1 mm × 50 mm) using as aqueous mobile phase a 0.01% formic acid solution (A) and acetonitrile (B) as organic modifier at a flow rate of 0.4 mL/min. The mobile phase composition was 100% A for the first 0.3 min, and then B was linearly increased until 100% in minute 3; this composition was held for 0.5 min. Then, it was returned to starting conditions in 0.1 min, and the system was equilibrated until minute 5. The injection volume was 5 μL, and the autosampler temperature was 10 °C.

### Postcolumn Infusion Conditions and Quality Monitoring

Isotopically labeled compounds atenolol-*d*_7_, caffeine-*d*_3_, diclofenac-^13^C_6_, lacidipine-^13^C_8_, metformin-*d*_6_, nifedipine-*d*_6_, and simvastatin-*d*_6_ were purchased from Alsachim, and acetaminophen-*d*_4_ was purchased from Sigma-Aldrich. For the postcolumn infusion, the IntelliStart pumping system of the Synapt G2-S instrument was used. A postcolumn infusion solution (10 μL/min) was combined with the LC flow after the chromatographic separation.

The postcolumn infusion standards were chosen based on their physicochemical properties. In this sense, a broad polarity range was covered, and the molecules show different MS ionization behaviors forming protonated molecular ions, Na^+^ and K^+^ adducts, or in-source fragments. In the work we present here, isotopically labeled compounds were employed as a proof of concept, but other compounds can be employed for the same purpose.

The concentration of the standards in the postcolumn infusion solution was optimized in order to avoid ion suppression problems if the concentration was too high or the effect of the background noise if it was too low. In this way, the concentrations chosen for the postcolumn infusion solution were 0.025 mg/L atenolol-*d*_7_, 0.125 mg/L caffeine-*d*_3_, 0.25 mg/L diclofenac-^13^C_6_, 0.030 mg/L lacidipine-^13^C_8_, 0.030 mg/L metformin-*d*_6_, 0.125 mg/L nifedipine-*d*_6_, 0.125 mg/L simvastatin-*d*_6_, and 0.25 mg/L acetaminophen-*d*_4_.

The postcolumn infusion of these standards allows us to acquire several matrix effect profiles by extracting the ion chromatograms for the protonated molecular ions (and/or Na^+^, K^+^ adducts and in-source fragments). These profiles can be explored to look for areas with significant ion suppression/enhancement, especially if profiles of the same extracted ion are overlaid and compared between samples or against a reference profile such as a solvent sample.

## Results and Discussion

### Evaluation of Sample Preparation

Because of the intrinsic selectivity of LC-MS, the main aim of sample preparation is not usually to remove compounds with same *m*/*z* or MS/MS transition but to reduce ion suppression. In this respect, postcolumn infusion can offer useful information about the efficiency of a sample preparation to remove compounds from the matrix. A common approach to quantify the matrix effect of an analytical method is to compare the responses of the analytes with and without a matrix.^13^ Although this approach is very valuable from a quantitative perspective, it only offers information about the matrix effect at certain retention times. By using postcolumn infusion the impact of the sample preparation in the whole chromatogram can be evaluated.^14^

**Figure 1:**
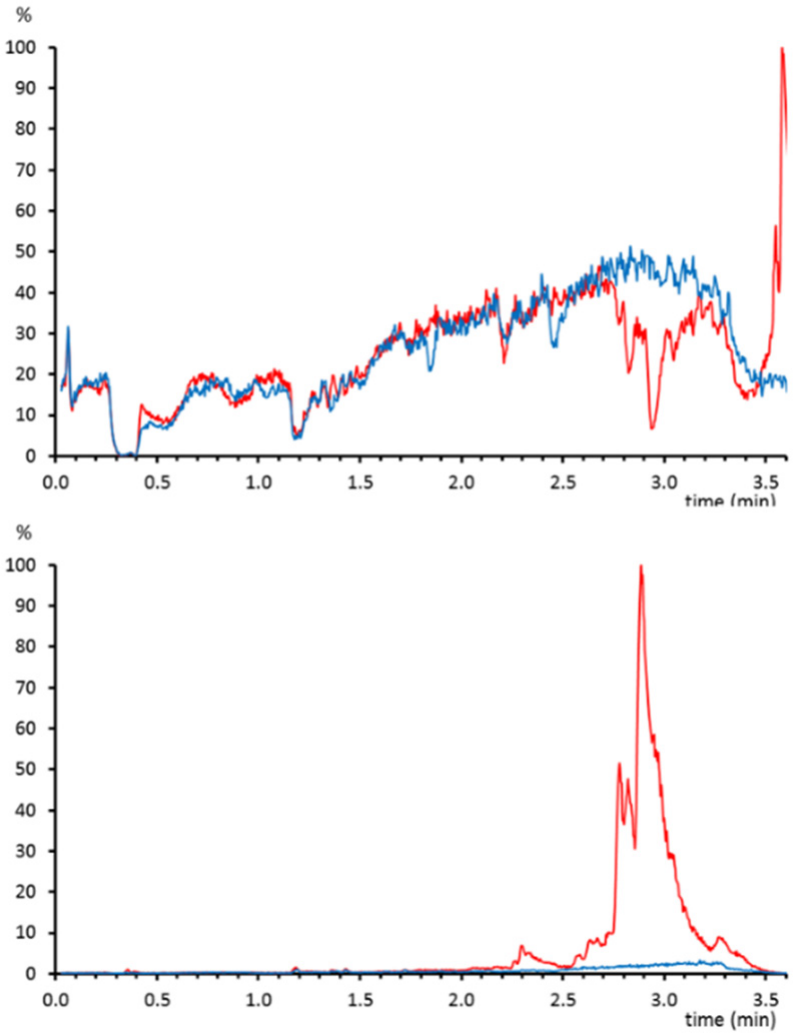
(above) Matrix effect profile for atenolol-*d*_7_ on a sample that was treated only with protein precipitation (red) or with phospholipid removal (blue). (below) Chromatogram of the phosphocholine fragment (184.075 *m*/*z*) extracted from the same samples.

This procedure was applied to study the efficiency of phospholipid removal cartridges (Ostro, Waters) from plasma samples. Plasma was spiked before and after treatment with several analytes with different physicochemical properties in order to calculate the matrix effect.^12^ After protein precipitation with acetonitrile in a 1:3 ratio, samples were either brought to dryness and reconstituted in mobile phase/methanol (55:45) or treated with the phospholipid removal cartridges before the evaporation and reconstitution step. In samples without a phospholipid removal step, late-eluting compounds (∼3 min) showed a high matrix effect. The quantitative values matched with the matrix effect profiles for the postcolumn infusion standards where a significant ion suppression area can be appreciated from 2.75 to 3.25 min (Figure 1).

For reversed-phase LC, these suppression areas are characteristic of nonselective protein precipitation sample treatments and are attributed to the elution of nonpolar compounds in plasma, mainly phospholipids. To confirm the presence of phospholipids, the characteristic ion fragment of phosphocholine (184.075 *m*/*z*) was extracted from the high collision energy scan function (Figure 1). Not surprisingly, the abundance of phospholipids was much higher in the samples in which only protein precipitation was performed. In this way, the satisfactory removal of compounds causing ion suppression was easily evaluated by means of postcolumn infusion.

### Identification of Nonexpected Sources of Matrix Effect

**Figure 2:**
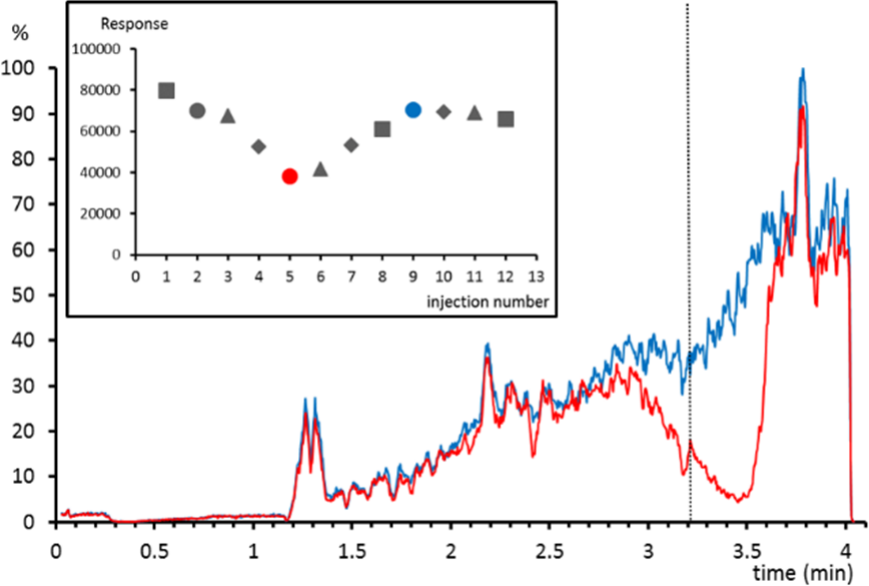
Matrix effect profile for simvastatin-*d*_6_ in a sample where a low response for simvastatin was observed (red) and in a sample where the expected response was observed (blue). Dotted line indicates the retention time of simvastatin. (inset) Response of simvastatin for urine samples (circles, triangles, and diamonds) or standard solutions (squares) spiked at 0.05 mg/L.

*Chromatographic buildup of phospholipids.* Three replicates of three different urine samples spiked with 0.05 mg/L of simvastatin (lipophilic cardiovascular drug) were diluted (1:10) and analyzed with the LC-MS method. Taking into account the late retention time of the drug, a low matrix effect was expected, since very nonpolar metabolites are scarce in urine. Surprisingly, not only the intragroup precision was poor (relative standard deviation (RSD) 22.3%) but also the intergroup precision (RSD: 16.4–31.6%). Neither the sample preparation nor the endogenous urine compounds could explain the variability among samples spiked at the same concentration level. When matrix effect profiles were studied for those samples with a lower response, a highly suppressed area was observed at the retention time of simvastatin (3.2 min). It was also observed that the response of simvastatin showed a clear trend when plotted against the injection order: the response decreased after a few injections and returned to the initial values after a few others (Figure 2).

In order to investigate the cause of the ion suppression, the spectra of the suppressed areas in the LC-MS chromatograms were explored. By checking the databases, numerous *m*/*z* of endogenous lipids were identified as putative coeluting compounds. The existence of lipids was supported by the presence of the characteristic phosphocholine fragment in the high collision energy mass function that perfectly fit with the shape of the matrix profile suppression area. The presence of phospholipids was attributed to the injection of plasma samples in a previous analysis batch for which the same column was used. It is likely that some of those compounds remained in the column and eluted after a few injections causing uncontrolled ion suppression.^15^ It is important to highlight that a similar suppression was observed with all the postcolumn infusion standards, which suggests that phospholipids affect the ionization of the studied compounds in a similar way. In this way, it was proven that postcolumn infusion can be an easy way to detect unexpected suppression of the analytes that can lead to an unreliable quantification, as in this case.

#### Effect of Anticoagulant in the Ion Suppression

**Figure 3:**
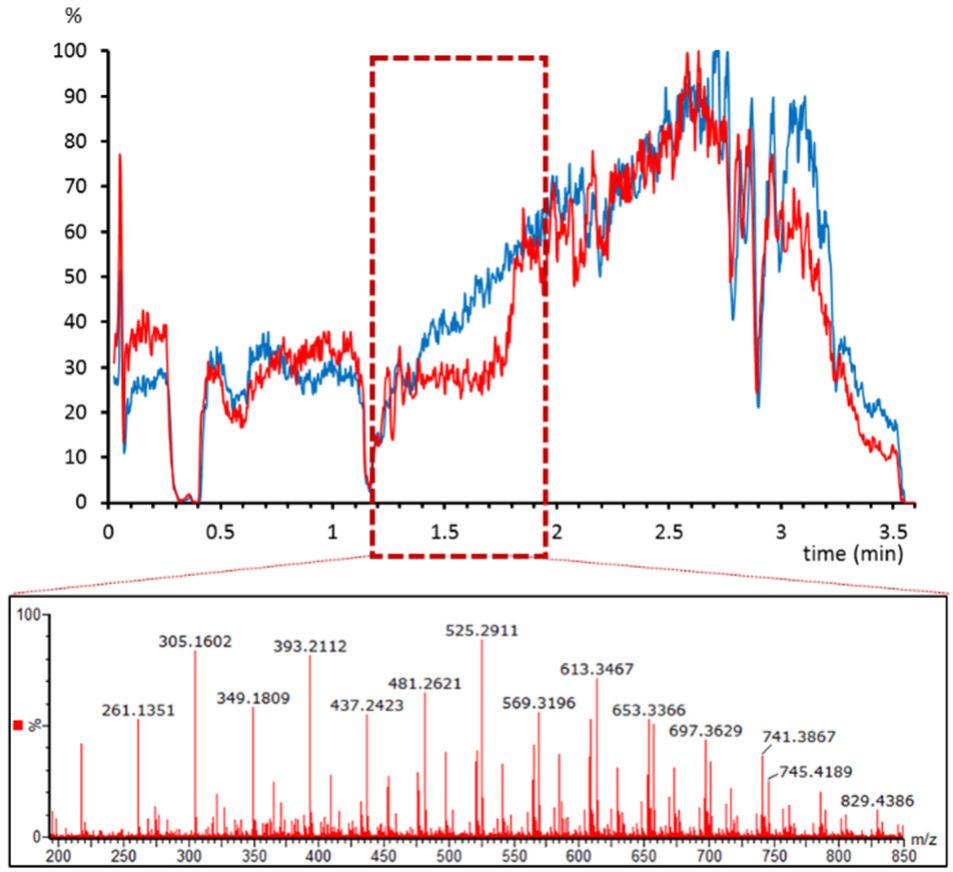
Matrix effect profile for atenolol-*d*_7_ in plasma samples stored in citrate (blue) and heparin (red) tubes. The box indicates the suppression area in heparin tubes. Below, the spectra extracted from that area show the characteristic 44 Da fragmentation of poly(ethylene glycol).

The choice of anticoagulant for plasma containers can be a critical factor in bioanalysis. Indeed, bioanalytical method validation guidelines require at least a partial validation when changing the anticoagulant.^16^ A set of plasma samples that had been stored in citrate or heparin containers were analyzed with the LC-MS method, and a clear effect of the anticoagulant in the ion suppression was observed. Heparin containers showed a high suppression area at ∼1.5 min that was not observed in the citrate containers (Figure 3). We studied the spectrum at that retention time, and we observed the characteristic ion cluster of 44 Da difference attributed to poly(ethylene glycol). This kind of matrix effect has been previously reported to be originated by plastic polymers in heparin sample collection tubes.^17^ Thanks to the postcolumn infusion it was possible to detect a suppression phenomenon that would have been more difficult to detect by other means.

## Conclusions

In this Application Note we showed that postcolumn infusion can be very useful to monitor the performance of LC-MS-based analytical methods. We demonstrated the benefits of postcolumn infusion of standards during the development of sample preparation and during routine analysis of biofluids. In this respect, postcolumn infusion gives insight about each individual sample analysis across the whole chromatogram, helping to detect (and understand) anomalies that may hamper a reliable quantification. In this aspect, postcolumn infusion is a complementary approach to the addition of isotopically labeled internal standards during the sample treatment. The latter methodology has been demonstrated to be an excellent option to correct for matrix effects, but it only offers valuable information on the performance of the method at certain retention times. Furthermore, postcolumn infusion can be used to monitor the instrument performance during a batch and explore the variability between quality control (QC) replicates or the decay in sensitivity with time.

Although we show some specific cases identified during the application of an analytical method to urine and plasma samples, this methodology can be easily implemented on any LC-MS platform, especially if the analysis is performed on High-Resolution instruments. In this Application Note we employed nine postcolumn infusion standards, but a lower number could be used, especially to study those factors that seem to be less analyte-dependent, such as intensity decay during the analytical run. The use of isotopically labeled compounds as postcolumn infusion standards is an interesting option considering they will rarely interfere with the analytes. Nevertheless, other (less expensive) compounds, like structural analogues, can be employed, if they are carefully chosen. Another critical parameter to be optimized is the concentration of the postcolumn infusion standards: too high concentrations can suppress the signal or affect the sensitivity of the method, and too low concentrations can hinder the monitoring of the postcolumn infusion standard signal. Overall, the use of postcolumn infusion of standards has the potential to improve the robustness of analytical methods as well as the reliability of the results they provide.
